# IL-6 expression helps distinguish Castleman’s disease from IgG4-related disease in the lung

**DOI:** 10.1186/s12890-021-01603-6

**Published:** 2021-07-10

**Authors:** Yasuhiro Kinugawa, Takeshi Uehara, Mai Iwaya, Shiho Asaka, Shota Kobayashi, Tomoyuki Nakajima, Masamichi Komatsu, Masanori Yasuo, Hiroshi Yamamoto, Hiroyoshi Ota

**Affiliations:** 1 Department of Laboratory Medicine, Shinshu University School of Medicine, 3-1-1 Asahi, Matsumoto, 390-8621 Japan; 2 First Department of Internal Medicine, Shinshu University School of Medicine, Matsumoto, Japan; 3 Department of Biomedical Laboratory Medicine, Shinshu University School of Medicine, Matsumoto, Japan

**Keywords:** Multicentric Castleman’s disease, IgG4-related disease, IgG4-related lung disease, Interleukin-6, RNAscope, RNA in situ hybridization

## Abstract

**Background:**

It is difficult to distinguish between multicentric Castleman’s disease (MCD) and IgG4-related lung disease (IgG4-LD), an IgG4-related disease (IgG4-RD) in the lung.

**Methods:**

We focused on IL-6, which is elevated in MCD, to distinguish between MCD and IgG4-LD by RNAscope, a highly sensitive RNA in situ method. Six cases of MCD and four cases of IgG4-LD were selected.

**Results:**

In all cases of MCD and IgG4-LD, 10 or more IgG4-positive cells were found in one high-power field. All MCD cases were inconsistent with the pathological IgG4-related comprehensive diagnostic criteria, but 2 of 6 cases had an IgG4/IgG ratio greater than 40%. In all IgG4-LD cases, histological features were consistent with the pathological IgG4-RD comprehensive diagnostic criteria. *IL-6* expression was observed in all MCD and IgG4-LD cases except for one IgG4-LD biopsy. *IL-6*-expressing cells were mainly identified in the stroma. Sites of *IL-6* expression were not characteristic and were sparse. *IL-6* expression tended to be higher in MCD compared with IgG4-LD. A positive correlation was found between the *IL-6* H-score and serum IL-6 level.

**Conclusion:**

Differences in *IL-6* expression may help distinguish between MCD and IgG4-LD. In addition, the presence of high IL-6 levels may help elucidate the pathological mechanisms of IgG4-LD.

## Background

IgG4-related disease (IgG4-RD) is characterized by mass lesions, elevated serum IgG4, IgG4-positive lymphoplasmacytic infiltration into affected organs, and fibrosis termed storiform fibrosis. Recently, cases of increased serum IgG4 [1, 2] and IgG4-positive plasma cell infiltration in damaged tissues [3, 4] were reported in diseases other than IgG4-RDs. Therefore, these phenomena may lead to the misdiagnosis of IgG4-RD and non-IgG4-RD. Multicentric Castleman’s disease (MCD) causes serum IgG4 elevation and IgG4-positive plasma cell infiltration despite being a non-IgG4-RD [5, 6]. MCD is a lymphoproliferative disorder caused by the hypersecretion of IL-6, resulting in polyclonal antibody production and plasma cell differentiation. Symptoms of MCD include fever, general malaise, loss of appetite, weight loss, and rash. In addition, MCD cases have abnormal laboratory values for anemia, elevated CRP, hypoalbuminemia, and hypergammaglobulinemia. MCD was reported to occasionally cause elevated serum IgG4 and IgG4-positive plasma cell infiltration. Steroids are used to treat IgG4-RD, whereas tocilizumab, an IL-6 receptor antibody, is used to treat MCD [7]. IgG4-RD and MCD are treated differently, despite similarities including mass lesions, elevated serum IgG4, and pathological findings such as IgG4-positive plasma cell infiltration. Therefore, the importance of differentiating between these diseases are described in the comprehensive diagnostic criteria for IgG4-RD [8].

Furthermore, reports of MCD and IgG4-related lung disease (IgG4-LD), an IgG4-RD and IgG4-related respiratory disease in the lung [9], both of which have interstitial lung lesions, have been increasing in recent years. However, there have been few clinical pathological comparative studies on lung lesions of MCD and IgG4-LD. High IL-6 levels are characteristic of MCD, but there have been few reports of its expression in tissues. Therefore, we analyzed the expression of IL-6 in MCD and IgG4-LD using high-sensitivity RNA in situ, and compared the findings between diseases clinicopathologically.

## Methods

### Patients and materials

This study was conducted in accordance with the Declaration of Helsinki and was approved by the ethics committee of Shinshu University School of Medicine (approval no. 5058). At Shinshu University Hospital, Matsumoto, Japan between 2008 and 2020, specimens of IgG4-LD and MCD in lung were selected.

Six MCDs were selected, three of which were resected specimens and three of which were biopsy specimens. Four cases of IgG4-LD were selected, two of which were resected specimens and two of which were biopsy specimens. Clinicopathological data were obtained from medical records. Materials used for evaluation were archived formalin-fixed paraffin-embedded tissues. Two pathologists (T.U. and M.I.) re-evaluated the histological features of all specimens.

### *IL-6* RNA in situ hybridization

*IL-6* mRNA was detected using an RNAscope kit (Advanced Cell Diagnostics, Hayward, CA, USA), as previously described [10]. Intracellular brown dots indicated positive staining. *IL-6* expression was quantified on the basis of a five‐grade scoring system recommended by the manufacturer (0, no staining; 1, 1–3 dots/cell; 2, 4-10 dots/cell; 3, > 10 dots/cell; and 4, > 15 dots/cell with > 10% of dots in clusters). The H‐score was calculated as: (% of grade 1 cells × 1) + (% of grade 2 cells × 2) + (% of grade 3 cells × 3) + (% of grade 4 cells × 4). The overall H‐score for each patient was calculated on the basis of the H‐score per high‐power field (400× magnification).

### Statistical analysis

Pearson’s chi-squared test, Wilcoxon rank sum test, and Spearman rank correlation test were analyzed by JMP Statistics software version 13 (JMP, Tokyo, Japan). A *P* value less than 0.05 was considered statistically significant.

## Results

### Clinicopathological features

Table 1 shows the clinicopathological features of the study samples. The serum IgG4 level was higher than the cut-off value (135 mg/dL) in all cases of MCD and IgG4-LD. There was no significant difference in serum IgG4 levels between the two groups.

**Table 1:** Clinicopathological data in MCD and IgG4-LD

	Diagnosis	Material	Age	Sex	Serum IgG4 (mg/dL)	Serum IL-6 (pg/mL)	*IL-6* H-score	IgG4/IgG ratio	Past histoly and comorbidities	Corticosteroid treatment	Tocilizumab treatment
1	MCD	Resected	34	M	290	99.4	12.16	31.3	Hepatosplenomegaly	No	Yes*
2	MCD	Resected	40	M	961	na	23.68	29.3	None	No	No
3	MCD	Resected	43	M	490	25.3	10.66	29.4	Cardiac hypofunction, diabetes mellitus	Yes*	No
4	MCD	Biopsied	46	F	318	26.6	na	14.0	None	No	No
5	MCD	Biopsied	65	F	147	10.1	3.70	57.1	Thymoma	No	No
6	MCD	Biopsied	40	M	269	17.9	11.59	59.1	None	Yes	Yes*
7	IgG4-LD	Resected	66	M	227	3.22	7.76	70.5	Diabetes mellitus	No	No
8	IgG4-LD	Resected	78	M	322	1.76	10.43	59.5	Emphysema, pulmonary fibrosis	No	No
9	IgG4-LD	Biopsied	71	F	1714	1.83	3.45	93.8	IgG4-RD (peritoneum and left orbit)	Yes*	No
10	IgG4-LD	Biopsied	67	M	605	0.523	0.00	91.1	IgG4-RD (pancreas and bile duct)	Yes*	No

*M* male, *F* female, *P* present, *A* absent, *na* not available

^*^After biopsy

### Histopathologic characteristics and immunohistochemical findings

**Fig. 1:**
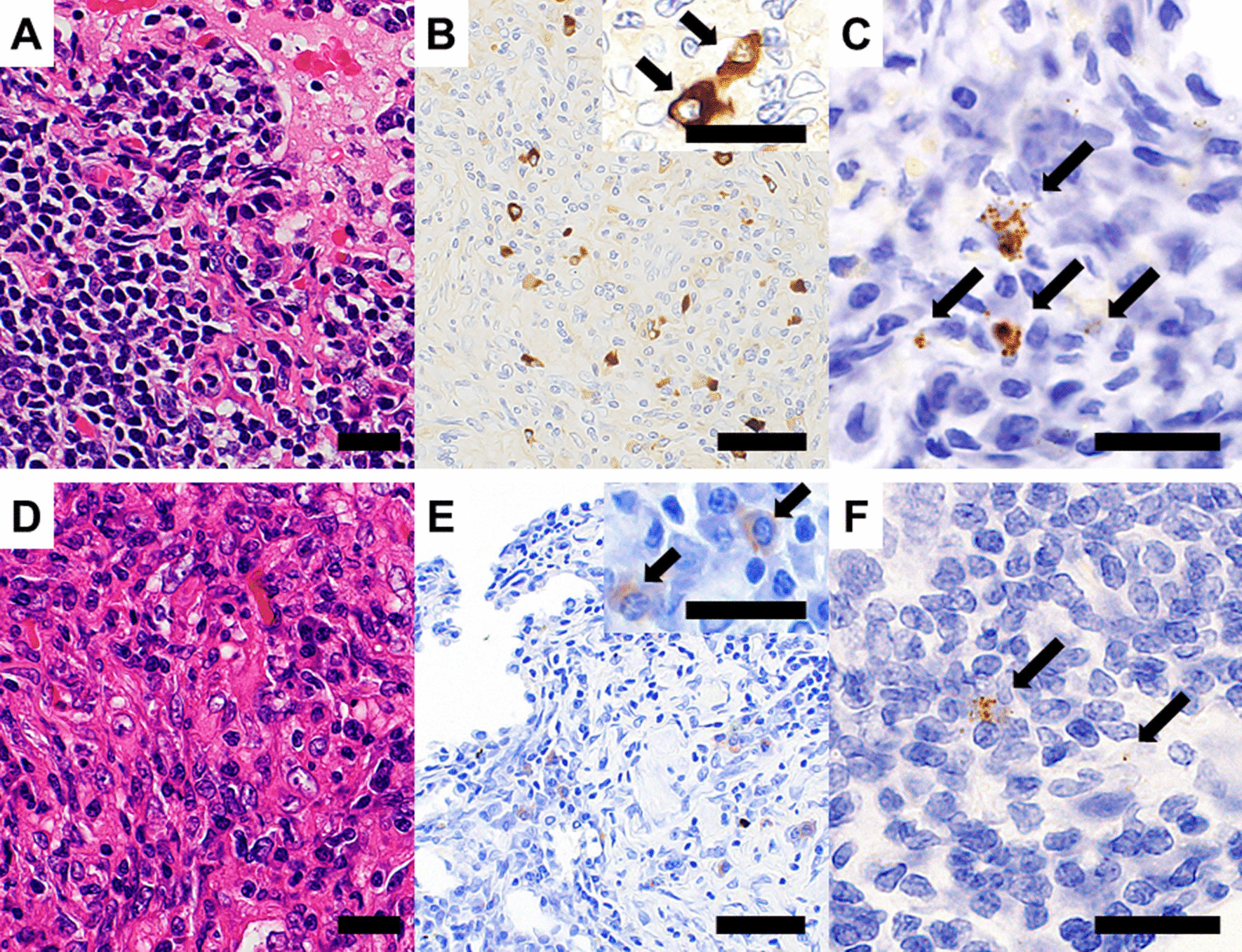
Representative histopathological images of IgG4 and *IL-6* expression. **a** Representative histopathological image of MCD. **b** Representative features of IgG4 expression in MCD. Insert shows a higher magnification image. Many IgG4-positive plasma cells (arrow) are identified. **c** Representative features of *IL-6* expression (arrow) in MCD. **d** Representative histopathological image of IgG4-LD. **e** Representative features of IgG4 expression in IgG4-LD. Insert shows a higher magnification image. Many IgG4-positive plasma cells (arrow) are identified. **f** Representative features of *IL-6* expression (arrow) in IgG4-LD. In **a**–**f**, the scale bar indicates 20 μm. Scale bar indicates 50 μm in the inserts

Lymphoplasmacytic infiltration was observed in MCD and IgG4-LD (Fig. 1a, d). However, storiform fibrosis was identified in 3 of 4 cases of IgG4-LD and none of the MCD cases. In all cases of MCD and IgG4-LD, 10 or more IgG4-positive cells were present in one high-power field (Fig. 1b, e). All MCD cases were inconsistent with the pathological IgG4-related comprehensive diagnostic criteria, but 2 of 6 cases had an IgG4/IgG ratio greater than 40%. In all IgG4-LD cases, histological features were consistent with the pathological IgG4-related disease comprehensive diagnostic criteria [8]. Lymphoid follicle formation was observed in 3 MCD resected samples, and none of the IgG4-LD cases.

### *IL-6* expression

*IL-6* mRNA in situ was performed, but one case with MCD was excluded due to negative internal control. *IL-6* expression was identified in all MCD and IgG4-LD samples except for one IgG4-LD biopsy (Fig. 1c, f). *IL-6*-expressing cells were mainly identified in the stroma. *IL-6* expression sites were not characteristic and were sparse. *IL-6* expression tended to be higher in MCD compared with IgG4-LD (Table 2).

**Table 2:** Clinicopathological characteristics of MCD and IgG4-LD

Factors	MCD (n = 6)	IgG4-LD (n = 4)	*P* value
Age	41.5 (38.5–50.75)	69 (66.25–76.25)	0.0139*
Sex (male/female)	4/2	3/1	0.7782
Serum IgG4 (mg/dL)	304 (238.5–607.75)	463.5 (250.75–1436.75)	0.4555
Serum IL6 (pg/mL)	25.3 (14–63)	1.75 (0.83225–2.8725)	0.0200*
*IL-6* H-score	11.59 (7.18–17.92)	5.605 (0.8625–9.7625)	0.0662
IgG4/IgG ratio	30.35 (25.475–57.6)	80.8 (62.65–93.125)	0.0142*

Data are presented as the median with 25th and 75th percentiles

^*^Significant *P* value < 0.05

### Association between *IL-6* expression and clinicopathological characteristics

Although the number of cases studied was limited, a positive correlation was found between the IgG4/IgG ratio and serum *IL-6* level, and between the IgG4/IgG ratio and *IL-6* H-score (Table 3). A positive correlation was also found between the serum *IL-6* level and *IL-6* H-score (Table 3).

**Table 3:** Correlation between serum IgG4, serum IL-6, *IL-6* H-score, and IgG4/IgG ratio

	Serum IgG4 (mg/dL)	Serum IL-6 (pg/mL)	*IL-6* H-score	IgG4/IgG ratio
Serum IgG4 (mg/dL)				
Spearman				
*P* value				
Serum IL-6 (pg/mL)				
Spearman	− 0.3667			
*P* value	0.3317			
*IL-6* H-score				
Spearman	− 0.05	0.8095		
*P* value	0.8984	0.0149*		
IgG4/IgG ratio				
Spearman	0.1273	− 0.85	− 0.8167	
*P* value	0.7261	0.0037*	0.0072*	

Spearman, Spearman’s rank correlation coefficient

^*^Significant *P* value < 0.05

## Discussion

In this study we demonstrated by RNA in situ hybridization (RNAscope) that *IL-6* mRNA-expressing cells were mainly present in the stroma both in MCD and in IgG4-LD, and that *IL-6* expression tended to be higher in MCD compared with IgG4-LD, which may help distinguish MCD from IgG4-LD.

Because *IL-6* expression is an important factor in MCD and is related to symptoms [6], discoveries of *IL-6* expression in tissues may be useful for the accurate diagnosis of MCD. Although *IL-6* expression was also observed in IgG4-LD, there was a relatively different *IL-6* H-score between MCD and IgG4-LD; therefore, expression analysis by RNAscope may be useful for differentiating between MCD and IgG4-LD, especially when serum IL-6 data are not available.

There have been several reports of increased IgG4-positive plasma cells by the histopathological analysis of clinicopathologigally diagnosed with MCD [6, 11]. In these reports, serum IgG4 levels were also elevated. Furthermore, high serum IgG4 levels and numbers of IgG4-positive plasma cells were found in tissues of clinicopathologigally diagnosed with MCD in the lung [12, 13]. However, there is no clear explanation of the mechanism involved in the increase in IgG4-positive plasma cell numbers and serum IgG4 levels in MCD. However, it might be related to activation of the Th2 cascade, which induces the secretion of IgG4. Indeed, Th2 lymphocytes are induced in MCD [14, 15]. Analysis of the relationship between Th2 lymphocytes and IgG4 in MCD is warranted.

Clinicopathologically, increased IL-6 levels were reported in IgG4-RD [16] and IL-6 was elevated in IgG4-related dacryoadenitis and sialadenitis [17]. The reason for high IL-6 levels in IgG4-RD is not well understood, but IL-6 is thought to directly promote the development of fibrosis in damaged tissues [18, 19]. Zongfei et al. reported elevated levels of IL-6 and IL-6R in the serum and tissues of IgG4-RD patients and that serum IL-6 was positively correlated with ESR, CRP, and IgG4-RD responder index, but not with serum IgG4 [16]. In addition, Tsukuda et al. reported that in IgG4-RD, the high IL-6 group was older, with lower albumin levels, and higher CRP and AST levels [20]. Liver swelling and splenomegaly were also significantly more common. Serum IL-6 levels in IgG4-RD may be significantly correlated with clinical inflammatory parameters. In addition, Tsukuda et al. concluded that serum IL-6 levels may be associated with the spread of disease to the bile ducts, liver, and spleen [20]. Previous studies reported cases that met the diagnostic criteria for IgG4-RD in the lung but with high IL-6 levels [12]. Therefore, IL-6 may only be increased for a specific period during IgG4-RD or in an organ-specific manner.

The concept of an “MCD-like” subtype of IgG4-RD, which has the characteristics of MCD and IgG4-LD, has been proposed [12]. Further studies on the clinicopathological features of the mixed type are required. Some of these cases did not respond to steroid administration suggesting they might represent a heterogeneous disease group.

Several studies have reported IL-6 expression in MCD and IgG4-RD by immunostaining and RNA in situ [7, 21]. Although the detection sensitivity of proteins such as cytokines by IHC may be insufficient, the detection of mRNA may be an effective alternative because it is localized in cells [22]. Otani et al. reported MCD cases that were *IL-6* negative by the RNA in situ method [7]. In detail, only 1 out of 8 cases of MCD showed focal *IL-6* positivity. Another study reported that *IL-6* was positive in situ [23]. However, H-scores were not measured in either study and the RNA in situ method used was different from ours. The difference in stainability due to *IL-6* in situ depends on the conditions at the time of staining, including the number of storage years and storage conditions.

This study had some limitations. These diseases are rare and the number of MCD and IgG4-LD samples was low in this study; therefore, further case accumulation is desired. IL-6 is secreted by various immunocompetent cells including T/B lymphocytes, monocytes, fibroblasts, and endothelial cells; however, its role and secreting cell type in MCD are unknown [14]. Therefore, it will be necessary to clarify which cells secrete IL-6 in MCD. In addition, it is unclear which factors induce the elevation of IL-6 in MCD and IgG4-LD. Confirmation using RNA Next-Generation Sequencing will be necessary.

## Conclusion

Regardless of the amount of *IL-6* expression, *IL-6* was identified in the affected organs of MCD and IgG4-LD; therefore, differentiation between these diseases should be judged by factors other than *IL-6*. However, the strong expression of *IL-6* may be supporting evidence of MCD. Further investigation of elevated *IL-6* expression in IgG4-LD might help elucidate the pathophysiology of IgG4-LD.

## Data Availability

All data generated and analyzed during the current study are available from the corresponding author on reasonable request.

## Abbreviations

IgG4-RD: IgG4-related disease
MCD: Multicentric Castleman’s disease
IgG4-LD: IgG4-related lung disease
